# Analysis of the complete mitogenomes of three high economic value tea plants (Tea-oil Camellia) provide insights into evolution and phylogeny relationship

**DOI:** 10.3389/fpls.2025.1549185

**Published:** 2025-04-24

**Authors:** Heng Liang, Huasha Qi, Chunmei Wang, Yidan Wang, Moyang Liu, Jiali Chen, Xiuxiu Sun, Tengfei Xia, Shiling Feng, Cheng Chen, Daojun Zheng

**Affiliations:** ^1^ Institute of Tropical Horticulture Research, Hainan Academy of Agricultural Sciences, Haikou, China; ^2^ Sanya Institute, Hainan Academy of Agricultural Sciences, Sanya, China; ^3^ Key Laboratory of Tropic Special Economic Plant Innovation and Utilization, Haikou, China; ^4^ Key Laboratory of Tropic Special Economic Plant Innovation and Utilization, Hainan Academy of Agricultural Sciences, Haikou, China; ^5^ School of Life Sciences, Technical University of Munich, Freising, Germany; ^6^ School of Agriculture and Biology, Shanghai Jiao Tong University, Shanghai, China; ^7^ College of Life Science, Sichuan Agricultural University, Ya’an, Sichuan, China

**Keywords:** mitochondrial genome, Tea-oil Camellia, comparative genomics, homologous fragments, phylogeny

## Abstract

**Introduction:**

Tea-oil Camellia species play a crucial economic and ecological role worldwide, yet their mitochondrial genomes remain largely unexplored.

**Methods:**

In this study, we assembled and analyzed the complete mitochondrial genomes of *Camellia oleifera* and *C. meiocarpa*, revealing multi-branch structures that deviate from the typical circular mitochondrial genome observed in most plants. The assembled mitogenomes span 953,690 bp (*C. oleifera*) and 923,117 bp (*C. meiocarpa*), containing 74 and 76 annotated mitochondrial genes, respectively.

**Results:**

Comparative genomic analyses indicated that *C. oleifera* and *C. meiocarpa* share a closer genetic relationship, whereas *C. drupifera* is more distantly related. Codon usage analysis revealed that natural selection plays a dominant role in shaping codon bias in these mitochondrial genomes. Additionally, extensive gene transfer events were detected among the three species, highlighting the dynamic nature of mitochondrial genome evolution in Tea-oil Camellia. Phylogenetic reconstruction based on mitochondrial genes exhibited incongruence with chloroplast phylogenies, suggesting potential discordance due to hybridization events, incomplete lineage sorting (ILS), or horizontal gene transfer (HGT). Furthermore, we identified species-specific mitochondrial markers, which provide valuable molecular tools for distinguishing Tea-oil Camellia species.

**Discussion:**

Our findings enhance the understanding of mitochondrial genome evolution and genetic diversity in Tea-oil Camellia, offering essential genomic resources for phylogenetics, species identification, and evolutionary research in woody plants.

## 1 Introduction

**Figure 1:**
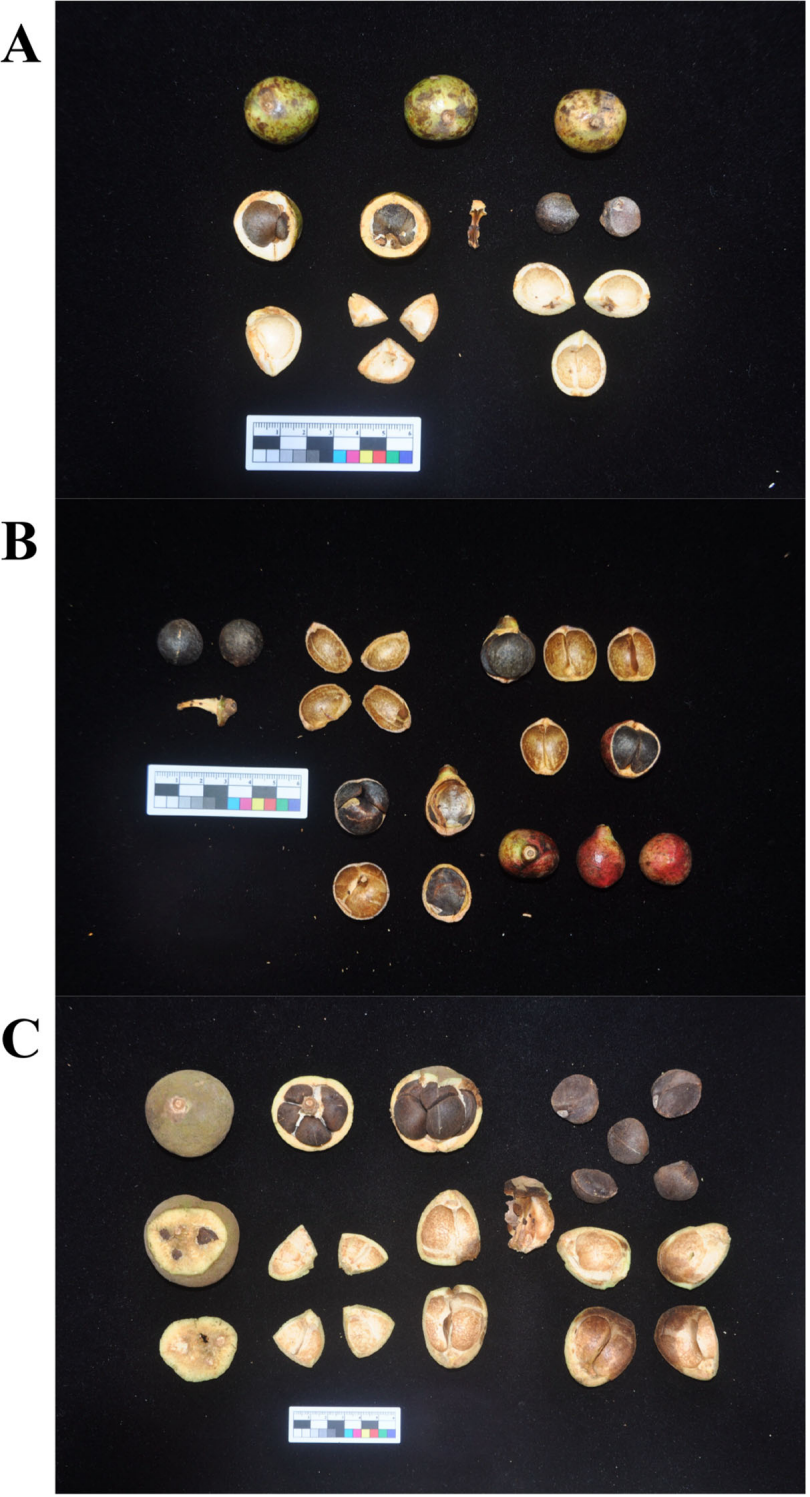
The morphology of three main Tea-oil Camellia (Camellia spp.) species fruit. **(A)** *C. oleifera*; **(B)** *C. meiocarpa* and **(C)** *C. drupifera*.

Tea-oil Camellia refers to a group of tree species within the *Camellia* genus of the Theaceae family. These are endemic economic forest tree species with a long history of utilization in China, comprising about 60 species (Yao and Ren, 2022). Currently, *C. oleifera* has the largest cultivation area, followed by *C. meiocarpa*, with *C. drupifera* ranking third (Zhu et al., 2024). These species represent three of the major Tea-oil Camellia crops in China (**Figure 1**). Among them, the cultivated *C. meiocarpa* is tetraploid, *C. oleifera* is primarily hexaploid, and *C. drupifera* is mainly octaploid (Qin et al., 2018). *C. drupifera* produces the largest fruits, while *C. meiocarpa* bears the smallest (Yao and Ren, 2022). The evolutionary dynamics of oil crops have long been a topic of interest, yet the phylogenetic relationships among *C. oleifera*, *C. meiocarpa*, and *C. drupifera* remain unclear due to frequent hybridization, apomixis, rapid radiation, and a rich history of diversity. Previous studies have attempted to classify these species based on morphological traits, cytological analyses, and molecular markers, but significant controversies persist (Qin et al., 2018; Qi et al., 2020, 2023). Besides that, reticulate evolution, driven by hybridization and introgression, plays a major role in shaping plant mitochondrial genomes. Unlike nuclear DNA, which undergoes biparental inheritance, mitochondrial DNA is maternally inherited and thus is particularly susceptible to cytoplasmic gene flow, horizontal gene transfer (HGT), and recombination events (Gogarten and Townsend, 2005; Soucy et al., 2015). Previous studies suggest that in *Camellia* species, mitochondrial genome restructuring may result from interspecific hybridization, leading to complex phylogenetic signals (Xiao et al., 2025). Additionally, the phenomenon of chloroplast-to-mitochondria gene transfer is frequently observed in angiosperms, influencing gene content and structural variation within mitochondrial genomes (Smith, 2014). However, in Tea-oil Camellia, whether these processes contribute to the observed phylogenetic discordance between mitochondrial and plastid genomes remains unclear.

In practical production, significant variations exist in both the quality and pricing of Tea oil derived from different species (Shi et al., 2020). Due to the difficulty in distinguishing between the three during the seedling stage, issues such as misuse, mislabeling, and substandard products are common, disrupting the tea-oil market (Liang et al., 2024). Therefore, understanding the phylogenetic relationships among the three major cultivated Tea-oil Camellia species and developing efficient methods for species identification have become urgent issues for the healthy development of the Tea-oil industry. Compared to single or limited gene fragments, organellar genomes contain a wealth of genetic information, providing numerous variable sites for plant phylogenetic studies (Wang et al., 2024). Specific molecular markers developed from organellar genomes can enable rapid identification of these species (Elyasigorji et al., 2023; Jo and Park, 2024). Additionally, the structural characteristics of mitochondrial genomes and gene transfer events between mitochondrial and chloroplast genomes hold significant importance in the study of speciation (Wang et al., 2007; Butenko et al., 2024; Liu et al., 2024a). Currently, most phylogenetic studies on major cultivated Tea-oil Camellia species have relied on single or limited universal fragment sequences, providing relatively few informative sites—some sequences even lack variation in certain species (Li et al., 2023). As a result, these studies may not accurately reflect the phylogenetic status of the major Tea-oil Camellia species.

Plant mitochondria are semi-autonomous organelles surrounded by two membranes, widely present in eukaryotic cells. They possess their own genetic material and genetic system, which are critical for energy conversion and function as the key site for oxygen-dependent respiration in eukaryotic cells (Møller et al., 2021; Wu et al., 2022). Unlike chloroplast genomes, the mitochondrial genome information available on the NCBI website is currently fewer than a thousand entries (as of November 01, 2024). Additionally, compared to the predominantly circular structure of chloroplast genomes, mitochondrial genomes exhibit a more complex range of structural features, including multipartite circular, Y-shaped, and H-shaped configurations (Robinson and Kunji, 2006). There are significant size variations in mitochondrial genomes among different plant species. Generally, mitochondrial genomes fall within the range of 200 kb to 3,000 kb, with the smallest terrestrial plant mitochondrial genome at about 66 kb and the largest at 11,300 kb (Sloan et al., 2012; Skippington et al., 2015). Even within the same plant species, mitochondrial genome sizes can vary significantly; for example, in the Cucurbitaceae family, the sizes of mitochondrial genomes among different species can differ by approximately sevenfold (Alverson et al., 2010). While nuclear and chloroplast genomes are widely used for species classification and phylogenetic analysis, mitochondrial genomes offer additional evolutionary insights that are not readily accessible from other organellar genomes (Cheng et al., 2025). For instance, mitochondrial genomes can reveal structural complexities and inter-organellar gene transfers that are critical for understanding the broader genomic dynamics within plant species (Kong et al., 2025).

Complete mitochondrial genomes offer distinct advantages in studies of speciation, population genetics, phylogenetics, and phylogeography (Liu et al., 2024; Wang et al., 2025). Mitochondrial genomes, unlike nuclear or chloroplast genomes, exhibit unique characteristics such as multi-branch structures, high recombination rates, and gene content variations that provide valuable insights into plant evolutionary processes. While nuclear and chloroplast genomes are widely used for species classification and phylogenetic analysis, mitochondrial genomes offer additional evolutionary insights that are not readily accessible from other organellar genomes. For instance, Tang et al. (2023) sequenced and characterized the mitochondrial genomes of 34 species from the Selaginellaceae family, focusing specifically on the *Selaginella* genus (Tang et al., 2023). They found that the mitochondrial genome structures were complex, with a positive correlation among GC content, mutation rates, and the quantity of RNA editing sites. The phylogenetic tree constructed in their analysis provided new insights into the evolutionary dynamics within the genus. Horizontal gene transfer is the process by which organisms acquire genetic material from unrelated species rather than through vertical inheritance (Kurland et al., 2003; Gogarten and Townsend, 2005; Soucy et al., 2015). In flowering plants, horizontal gene transfer (HGT) is uncommon in nuclear DNA but frequently occurs in mitochondrial genomes (Bock, 2010). In multicellular eukaryotes, HGT involving chloroplast and mitochondrial DNA can significantly influence gene content (Chang et al., 2013). Typically, in higher plants, DNA fragments migrate unidirectionally from chloroplasts to mitochondria, with few instances of transfer in the opposite direction (Smith, 2014). HGT disrupts reproductive barriers and complicates gene flow, making it a significant factor in molecular and macroevolution studies. In recent years, mitochondrial genome information has also been applied to research in plant phylogenetics. Yang et al. (2023) sequenced the mitochondrial genome associated with *Panax notoginseng*, constructed a phylogenetic tree to clarify the relationships among five medicinal species in the *Panax* genus, and developed specific molecular markers for their rapid identification based on intron region comparisons (Yang et al., 2023). Previous studies on Tea-oil Camellia mitochondrial genomes have focused primarily on single species or limited regions of the genome. For example, the mitochondrial genome of *C. drupifera* was recently assembled, revealing its multi-branch conformation, a structure that deviates from the commonly circular form observed in most plants (Liang et al., 2025). Similarly, the mitochondrial genomes of *C. oleifera* and *C. lanceoleos*a have been analyzed in other studies, but comprehensive comparisons and broader genomic surveys across main Tea-oil Camellia species are still lacking (Xiao et al., 2025). Furthermore, few have examined the extent of mitochondrial genome variation, genome rearrangements, or gene transfer events in Tea-oil Camellia species.

This study proposes the following research questions based on current studies and industry needs: (1) What are the structural characteristics of the mitochondrial genomes of the three main tea-oil Camellia species? (2) Compare phylogenetic relationships inferred from mitochondrial and chloroplast genomes to assess whether hybridization and incomplete lineage sorting (ILS) have influenced the evolutionary relationships among the Tea-oil Camellia species (3) Can mitochondrial genome information be used to develop specific molecular markers for these species? Through the study of these questions, we aim to reveal the structural features and variations of the mitochondrial genomes of three main Tea-oil Camellia species. Based on this information, we will construct a phylogenetic tree to infer their evolutionary relationships. Additionally, we will design specific molecular markers to lay the foundation for the in-depth identification, evaluation, and utilization of tea-oil resources, providing reliable genomic information for the assessment of other Tea-oil species. This study aims to address these gaps by providing a comprehensive assembly and analysis of the mitochondrial genome of major Tea-oil Camellia species. By integrating comparative genomics, phylogenetics, and molecular marker development, it offers new insights into their evolutionary history and genome dynamics, contributing essential resources for species identification and conservation efforts.

## 2 Materials and methods

### 2.1 DNA sequencing assembly and annotation of *C. oleifera* and *C. meiocarpa*

Young leaves of *C. oleifera* and *C. meiocarpa* collected from the Hainan Academy of Agricultural Sciences (HAAS) were used as materials for this study. Genomic DNA of high quality was isolated from the leaves using a modified CTAB method (Porebski et al., 1997). We employed a sequencing strategy that combined third-generation “ONT” with second-generation “Illumina” technologies to construct libraries. Following the construction of libraries with SQK-LSK109 (Oxford Nanopore Technologies, Cambridge, UK), DNA sequencing was carried out on the Nanopore PromethION platform, resulting in the generation of 23.17 Gb in *C. oleifera* and 21.32 Gb in *C. meiocarpa* of raw data. The data were subsequently filtered and refined using NanoFilt and NanoPlot tools from Nanopack (De Coster et al., 2018). At the same time, libraries with an average fragment length of 350 bp were created from the high-quality DNA using the NexteraXT DNA Library Preparation Kit. Sequencing was subsequently conducted on the Illumina Novaseq 6000 platform (Illumina, San Diego, CA, USA), generating 42.02 Gb in *C. oleifera* and 41.03 Gb in *C. meiocarpa* raw sequence data (Patel and Jain, 2012). The data were then processed using the NGS QC Tool Kit v2.3.3 for editing.

The assembly method for the mitochondrial genomes was as follows: (1). Use minimap2 to align the mitochondrial sequences among related species *C. drupifera* (Li, 2018). (2). Assemble the aligned data using Flye v 2.9.5 (Kolmogorov et al., 2019), followed by correction with Racon v1.4.3 (Vaser et al., 2017). After correction, align the corrected results with the second-generation sequencing data using Bowtie2 v2.5.4 (Langmead and Salzberg, 2012). (3). Perform hybrid assembly using Unicycler v0.5.1 (Wick et al., 2017), and finally, split the GFA file based on the coverage from the third-generation data for the purpose of obtaining the final assembly results by MUMmer v4.0.0 (Marçais et al., 2018).

The protein-coding genes (PCGs) and rRNA of the mitochondrial genome were identified using blastn (Alverson et al., 2010) against closely related species *C. drupifera*, while tRNA was identified via tRNA scan-SE software (http://lowelab.ucsc.edu/tRNAscan-SE/). The introns’ boundaries were manually checked and corrected to ensure that the protein-coding genes had a complete structure. OGDRAW was utilized to construct the mitochondrial genome map (Greiner et al., 2019).

### 2.2 Comparison and analysis of three main Tea-oil species mtDNA

The mitochondrial genome of *C. drupifera* used in this study was retrieved from the Genbank of NCBI (accession numbers: PQ041261 and PQ041262) (Liang et al., 2025). Using MUMmer v4.0.0 (Marçais et al., 2018) with the –maxmatch parameter, we conducted pairwise genomic alignments to generate a dot-plot. We performed a pairwise alignment of the mitochondrial genomes with BLASTn software (v2.7.1) (Chen et al., 2015), identifying homologous sequences among them while applying a minimum collinear block length cutoff of 500 bp. For visualization and collinearity analysis of the three main Tea-oil Camellia species, we utilized Mauve v2.4.1 software (Darling et al., 2004).

Using Phylosuite (Zhang et al., 2020) software, we extracted the PCGs of the three major tea-oil Camellia species and aligned the homologous sequences with MAFFT. After alignment, utilizing KaKs_Calculator v2.0 (https://sourceforge.net/projects/kakscalculator2/) with the MLWL method, we calculated the Ka and Ks values for each gene pair. We then computed the Ka/Ks ratios for each gene pair and visualized the results with a box plot. Additionally, we analyzed haplotype diversity (Hd) using DnaSP6 (Rozas et al., 2017) software, generating a nexus haplotype data file. Nucleotide diversity (Pi) values were also calculated with DnaSP6. Finally, we employed Rscript to illustrate the collinearity relationships among gene orders in the three Tea-oil Camellia species.

### 2.3 Repeat sequences of three main Tea-oil species

Repeated sequences are significant features of genomes that influence evolution, heredity, and variation (Jurka et al., 2007). These elements are critical for regulating gene expression, transcription processes, chromosome architecture, and physiological metabolism (Li et al., 2018). Simple sequence repeats (SSRs) were detected using MISA software with the following parameters: 1-10 2-5 3-4 4-3 5-3 6-3 (Beier et al., 2017). For tandem repeats, TRF software was employed (parameters: 2 7 7 80 10 50 2000 -f -d -m). Dispersed repeats were identified via BLASTn (v2.10.1) with parameters set to -word_size 7 and evalue 1e-5, while redundancies and tandem repeats were removed. The outcomes were visualized using Circos.

### 2.4 Investigating relative synonymous codon usage and effective codon count

MEGA software facilitated the analysis of codon usage and the determination of RSCU from the mitochondrial genomes’ protein-coding genes (Kumar et al., 2016). The coding genes’ GC content was evaluated with the CUSP tool (https://www.bioinformatics.nl/cgi-bin/emboss/cusp), and the ENC was derived using CodonW to assess the randomness of genomic codon usage (Sharp and Li, 1986). Using Microsoft Excel 2021, we count the frequency of the third base of the RSCU greater than 1 and generated a scatter plot in two dimensions.

### 2.5 Investigation of DNA migration from chloroplasts to mitochondria

Utilizing BLAST v2.7, homologous sequences between the chloroplast and mitochondrial genomes were identified, with an E-value threshold of 1e−5 and a minimum similarity of 70%.

### 2.6 RNA editing sites of three main Tea-oil species mtDNA

A previous study indicated that mitochondrial genes experience numerous RNA editing events (Shi et al., 2016). RNA editing sites were predicted in the shared PCGs of three major Tea-oil species, utilizing the PREP-Mt online server (http://prep.unl.edu/) with a threshold score set at C = 0.2 (Mower, 2005).

### 2.7 Construction of phylogenetic trees of three main Tea-oil species mtDNA

The phylogenetic tree analysis involved a total of 16 species with shared PCGs from both mitochondrial and chloroplast genomes, including two outgroup species. The best evolutionary model for the PCGs was determined using ModelTest-NG relying on the Akaike Information Criterion (AIC). We executed a maximum likelihood (ML) analysis of the two datasets was performed using RAxML v8.2.13 (Darriba et al., 2020), involving 1,000 rapid bootstrap resampling. Using MrBayes v3.2.7, Bayesian inference (BI) trees were generated through a Markov Chain Monte Carlo (MCMC) approach, totaling 1,000,000 generations, sampling every 100 generations, and applying a burn-in of 25% (Ronquist et al., 2012).

### 2.8 In silico analysis of molecular markers in three main Tea-oil species

The polymorphism of plant mitochondrial genomes provides a valuable tool for distinguishing closely related species. We used MUMmer4 (Marçais et al., 2018) with *C. oleifera* as the reference to detect SNPs and indels between genomes. Each identified variant has been compiled into a table to facilitate species identification and downstream analysis. Primer design was conducted using Primer3 (Untergasser et al., 2012) with the parameters set to PRIMER_PRODUCT_SIZE_RANGE=150-300 and PRIMER_MAX_END_STABILITY=250.

## 3 Results

### 3.1 The mitochondrial genome architecture of *C. oleifera* and *C. meiocarpa*

**Figure 2:**
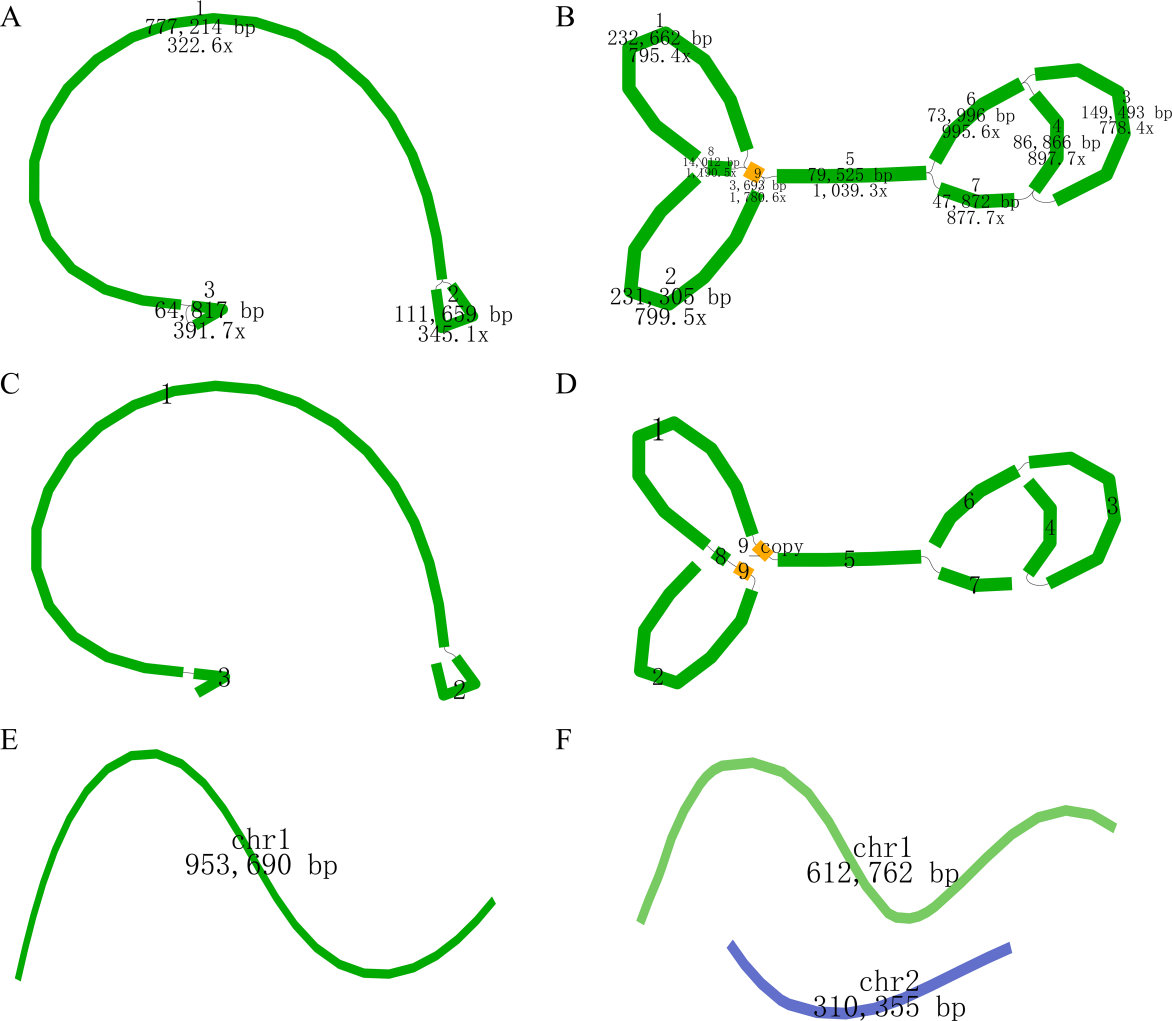
The assembly result of the mitochondrial genome of *C. oleifera* **(A, C, E)** and *C. meiocarpa* **(B, D, F)**. **(A, B)**: the original structure; **(C, D)**: re-drawing of **(B)**; **(E, F)**: the generated sequence in the end.

The newly sequenced genomes of *C. oleifera* and *C. meiocarpa* were reported (**Supplementary Tables S1**, **S2**). Based on the assembled results, the whole mitochondrial genomes of *C. oleifera* and *C. meiocarpa* were 953,690, and 923,117 bp, respectively (**Table 1**; **Figures 2A, B**). The GC content in their mitochondrial genomes were 45.68% (*C. oleifera*) and 45.73% (*C. meiocarpa*), aligning closely with the GC content found in mt genomes of other plant species that have been fully sequenced (Alvarenga et al., 2024; Liu et al., 2024b). The two Tea-oil Camellia species mitochondrial genome were found to possess peculiarities of multi-branch conformation (the same as another main Tea-oil Camellia species *C. drupifera*), which were not the typical circular structure. To present the structural and positional information of all genes, we linearized the branched genomes (**Figures 2C–F**). For convenience in detailing the genomic characteristics, we structured the assembly as one chromosome (three contigs—ctg3, ctg1 and ctg2) in *C. oleifera* and two chromosomes (seven contigs—ctg2, ctg9, ctg8, ctg1, ctg9_copy, ctg5 and ctg7; and three contigs—ctg6, ctg3 and ctg4) in *C. meiocarpa*. Through read mapping, we validated the quality of the assembly, confirming that all sites had adequate support from the reads. The depth was 332.6 to 391.7 in *C. oleifera* and 778.4.6 to 1780.6 in *C. meiocarpa*, respectively. To assess the quality and accuracy of our newly assembled *C. oleifera* mitochondrial genome, we compared it with the previously published mitochondrial genome (Xiao et al., 2025). Our new assembly, spanning approximately 86,148 bp, reveals structural features consistent with a multi-branch conformation, similar to those reported for other *Camellia* species (Liang et al., 2025; Xiao et al., 2025). This evidence further suggests that *Camellia* mitogenome deviates from the traditional circular structure and instead consists of multiple units.

**Table 1:** The assembly statistics in *C. oleifera* and *C. meiocarpa*.

	*C. oleifera*	*C. meiocarpa*
Number of contigs	3	9
Largest contig	777, 214	232,662
Smallest contig	64,817	3,693
Undetermined bases	None	None
GC content	45.68	45.73

**Figure 3:**
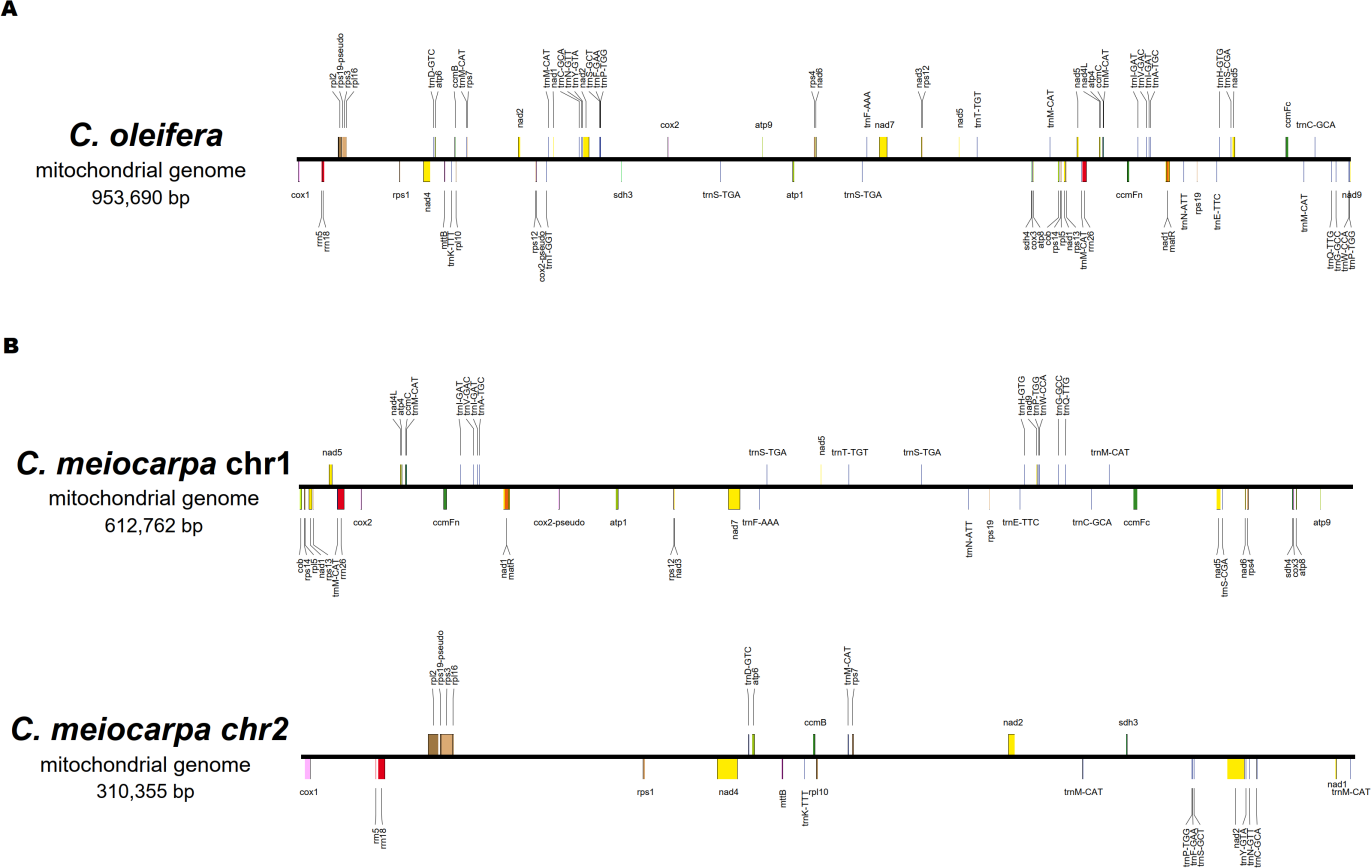
The order, orientation, and size of the genes within **(A)**: *C. oleifera* and **(B)** *C. meiocarpa* mitochondrial genome.

A total of 76 characteristic genes of the mitochondrial genome (39 PCGs, 32 tRNA genes, three rRNA genes and two pseudo genes) in *C. oleifera* and 74 (38 PCGs, 31 tRNA genes, three rRNA genes and two pseudo genes) in *C. meiocarpa* were annotated. For the purpose of comparing, we split the mitochondrial conformation into linear molecules. In *C. oleifera*, the majority Chromosome 1 encodes 76 genes (39 PCGs, 32 tRNA genes, three rRNA genes, and two pseudo genes). In *C. meiocarpa*, the majority Chromosome 1 encodes 47 genes (25 PCGs, 20 tRNA genes, one rRNA gene and one pseudo gene), and the minority Chromosome 2 encodes 27 genes (four PCGs, 13 PCGs, 11 tRNA genes, two rRNA genes and one pseudo gene) (**Figure 3**). Of the identified PCGs, 24 were classified as core genes, and 14 were considered non-core genes. The core gene set consists of five ATP synthase-related genes (*atp1*, *atp4*, *atp6*, *atp8*, *atp9*), nine genes related to NADH dehydrogenase (*nad1*, *nad2*, *nad3*, *nad4*, *nad4L*, *nad5*, *nad6*, *nad7*, *nad9*), four genes involved in cytochrome c biogenesis (*ccmB*, *ccmC*, *ccmFc*, and *ccmFN*), three cytochrome c oxidase genes (*cox1*, *cox2*, and *cox3*), one gene for a transport membrane protein (*mttB*), a maturase gene (*matR*), and a gene for ubiquinol-cytochrome c reductase (*cob*). Conversely, the non-core gene set includes four genes coding for large ribosomal subunits (*rpl10*, *rpl16*, *rpl2*, and *rpl5*), eight genes coding for small ribosomal subunits (*rps1*, *rps12*, *rps13*, *rps14*, *rps19*, *rps3*, *rps4*, and *rps7*), along with two succinate dehydrogenase genes (*sdh3* and *sdh4*). Most of gene arrangement of all the three main Tea-oil Camellia species were the same. Only the Gene dosage in *trnP-TGG*, *trnS-CGA*, *trnS-GCT* and *trnW-CCA* were different in three main Tea-oil Camellia species (**Supplementary Table S3**).

### 3.2 Comparison of the three main Tea-oil Camellia species mtDNA

**Figure 4:**
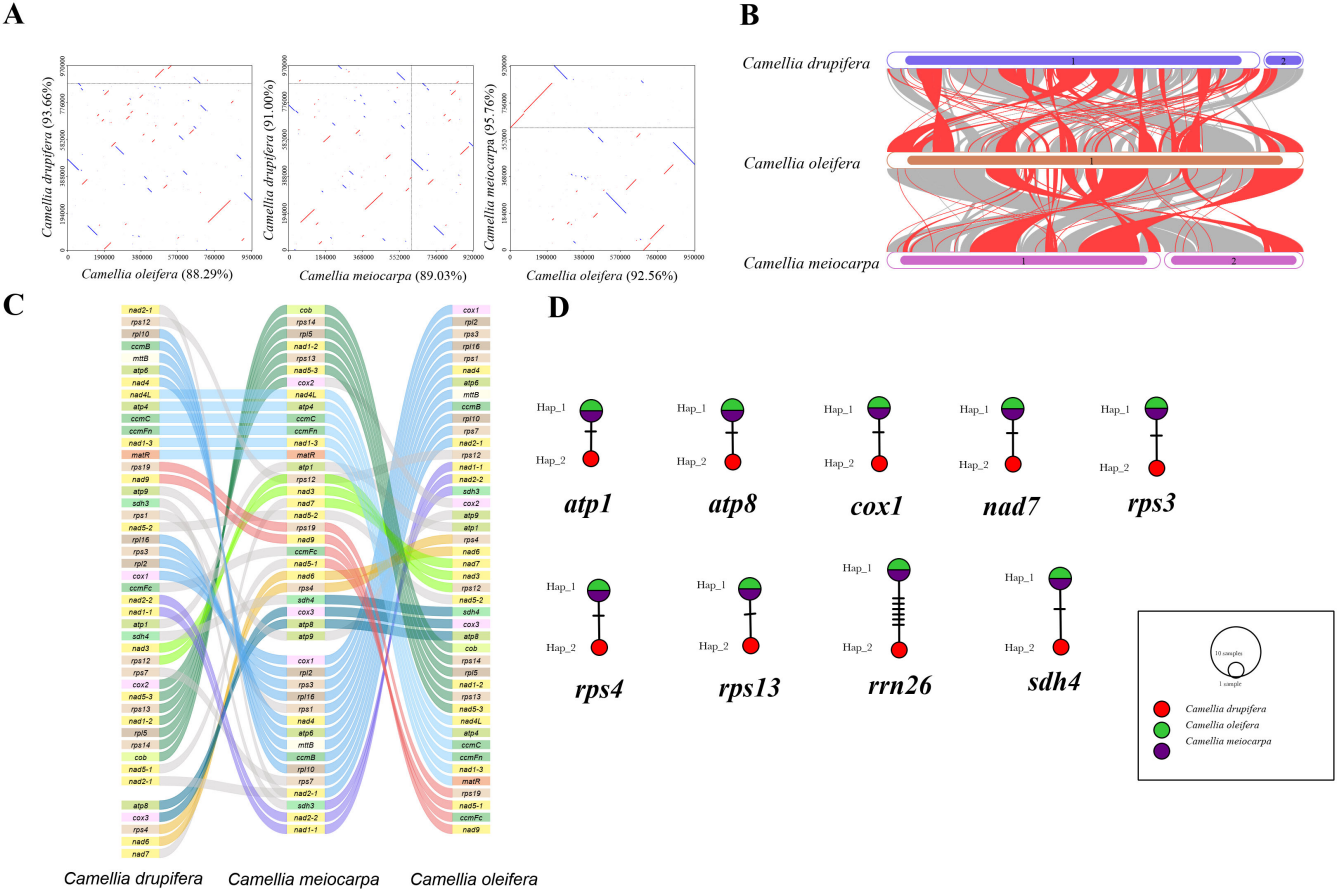
Summarizes the comparison of three main Tea-oil Camellia species. **(A)** Dot-plot graphs indicating synteny sequences. **(B)** Collinear analysis. **(C)** Gene order in the mitogenomes. **(D)** Haplotype networks of 9 shared genes.

For exploration of the evolutionary characteristics of mitochondrial genome of the three main Tea-oil Camellia species, we first compared them by synteny analysis. A visual representation of their relationships in **Figure 4A**. Dot-plot analysis revealed that *C. oleifera* and *C. meiocarpa* have longer and more similar synteny sequences compared to those found between *C. oleifera* and *C. drupifera*, and *C. meiocarpa* and *C. drupifera*. Pairwise synteny analysis identified numerous homologous collinear blocks, their arrangement varied across individual mitogenomes. These significant rearrangements suggest that the mitogenomes of the three main Tea-oil Camellia species exhibit considerable structural conservation. To further evaluate the connection among the three main Tea-oil Camellia species, the BLASTN program was used to compare homologous genes and their sequence organization. We specifically focused on conserved collinearity blocks of 500 bp or longer, retaining those exceeding 0.5 kb to clearly illustrate the collinear results. (**Figure 4B**). The plant mitogenomes displayed a dispersion of homologous sequences, particularly among closely related species, which shared the most homologous sequences. Moreover, collinear blocks across the mitochondrial genomes displayed irregular arrangements, with three main Tea-oil Camellia species showing multiple gene rearrangements that led to shorter collinear blocks. This suggested that while the mitochondrial genomic arrangement was highly conserved among three main Tea-oil Camellia species, they have also experienced the frequent gene recombination events.

We conducted synteny and rearrangement analyses among the three main Tea-oil Camellia species. Between the mitogenomes of *C. drupifera* and *C. meiocarpa*, eight conserved PCG segments were identified, including *rpl10 to nad4, nad4L to matR, rps19 to nad9, rpl16 to cox1, nad2-2 to nad1-1, nad3 to rps12, cox2 to cob, and atp8 to nad6* (**Figure 4C**). In the comparison between *C. meiocarpa* and *C. oleifera*, 11 conserved PCG segments were noted, encompassing *rpl15 to cob, sdh4 to atp8, nad1-1 to rps11, nad15-1 to rps12, rpl2 to rpl16, rps13 to nad1-2, nad1-4 to nad5-2, rps1 to rps7, matR to nad1-3, atp4 to nad4L, and cox2 to nad7*.

In addition, we looked at the mutation sites in the nine shared genes among the three main Tea-oil Camellia species to construct haplotype networks for a more in-depth analysis of genetic variation (**Figure 4D**). Notably, *C. drupifera* displayed the most distinct haplotype composition, whereas *C. oleifera* and *C. meiocarpa* shared an identical composition in these genes. Our analysis aimed to explore the phylogenetic relationships of three main Tea-oil Camellia species, the evolutionary characteristics of the three main Tea-oil Camellia species showed *C. oleifera* and *C. meiocarpa* have a close relationship then *C. drupifera*.

**Figure 5:**
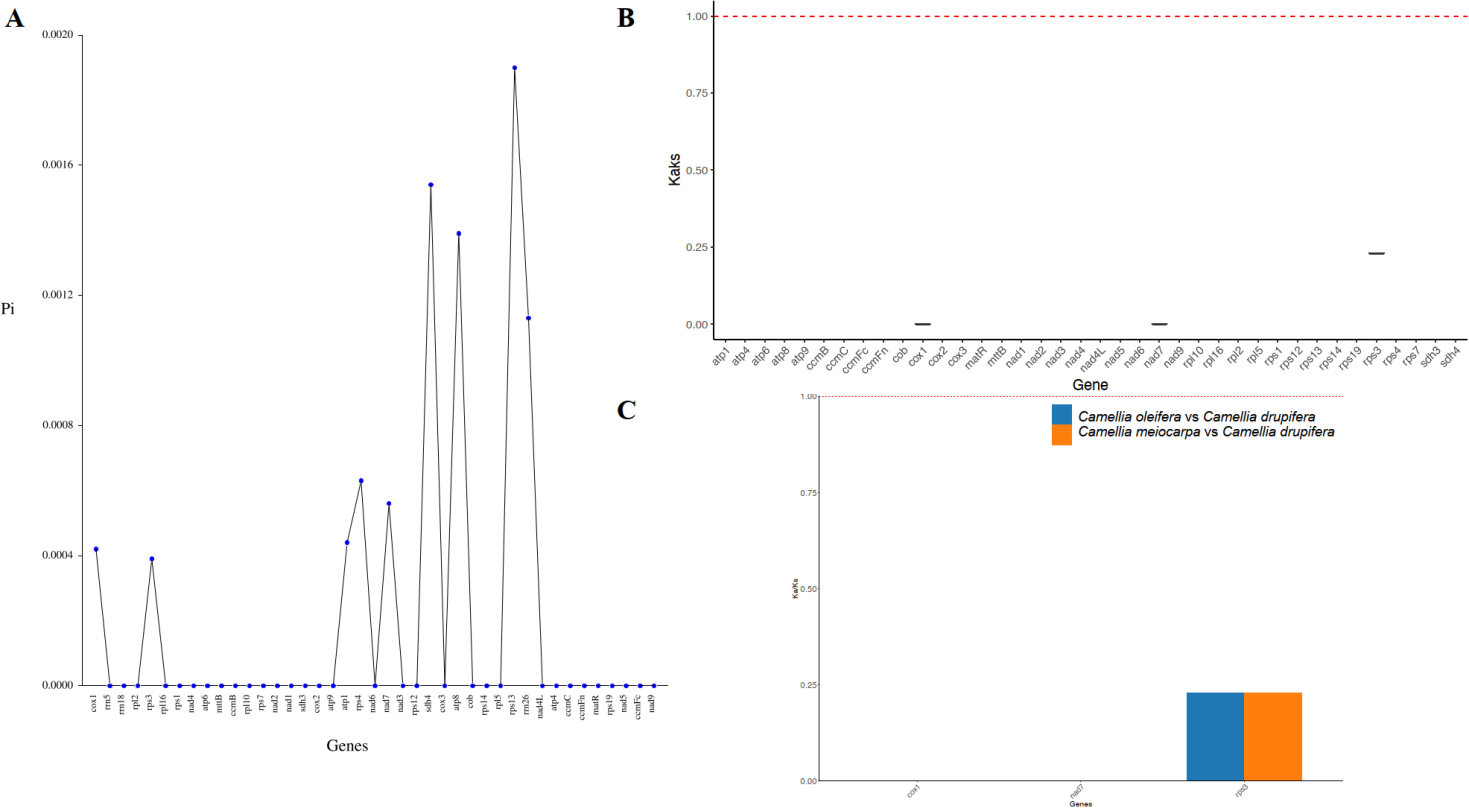
Variation in mitochondrial genes and the evolutionary characteristics of three main Tea-oil Camellia species. **(A)** nucleotide diversity of the PCGs; **(B, C)** Ka/Ks ratio calculated for the PCGs.

In order to assess the variation in PCGs and explore evolutionary patterns, we calculated the Pi and Ka/Ks ratios for each PCG across the three primary Tea-oil Camellia species, finding that the average Pi values ranged from 0 to 0.0019. The nine genes with the Pi values were *rps13* (0.0019), *sdh4* (0.00154), *atp8* (0.00139), *rrn26* (0.00113), *rps4* (0.00063), *nad7* (0.00056), *atp1* (0.00044), *cox1* (0.00042) and *rps3* (0.00039); the Pi values in most genes was 0, such as *mttB*, *ccmB*, *rpl10* et al. Patterns in genetic distance among PCGs were consistent, with *rps13* (0.0019), *sdh4* (0.00154), *atp8* (0.00139), and *rrn26* (0.00113) recognized as genes with rapid evolutionary rates than other PCGs, whereas *cox1* (0.00042), and *rps3* (0.00039) were slow evolving genes (**Figure 5A**; **Supplementary Table S4**). To assess the evolutionary rate of PCGs, *C. drupifera* as the reference, we calculated the average Ka/Ks ratio, which serves as a key metric for molecular adaptation. The Ka/Ks of most of genes were NA. The average Ka/Ks of three genes exist values, including *cox1*(Ka/Ks=0), *nad7* (Ka/Ks=0) and *rps3* (Ka/Ks= 0.23), which were consistently less than one (**Figures 5B, C**; **Supplementary Table S5**). The results showed that all genes have experienced relaxed selection and demonstrate minimal evolutionary change. These genes may be crucial for the adaptive processes of Tea-oil Camellia species.

Regions with significant nucleotide divergence are often utilized to create species-specific markers, particularly in closely related species that share similar morphological traits. While the *cox1* gene is commonly regarded as a universal DNA barcode for species identification and phylogenetic studies, this study revealed it to be the least variable and slowest evolving among the analyzed genes. In contrast, the *rps13*, *sdh4*, and *atp8* genes displayed the highest levels of variability and the fastest evolutionary rates. Consequently, these genes may serve as more suitable candidates for population genetic research and for distinguishing the three primary Tea-oil Camellia species.

### 3.3 Repetitive sequences present in the mitochondrial genome of three main Tea-oil Camellia species

**Figure 6:**
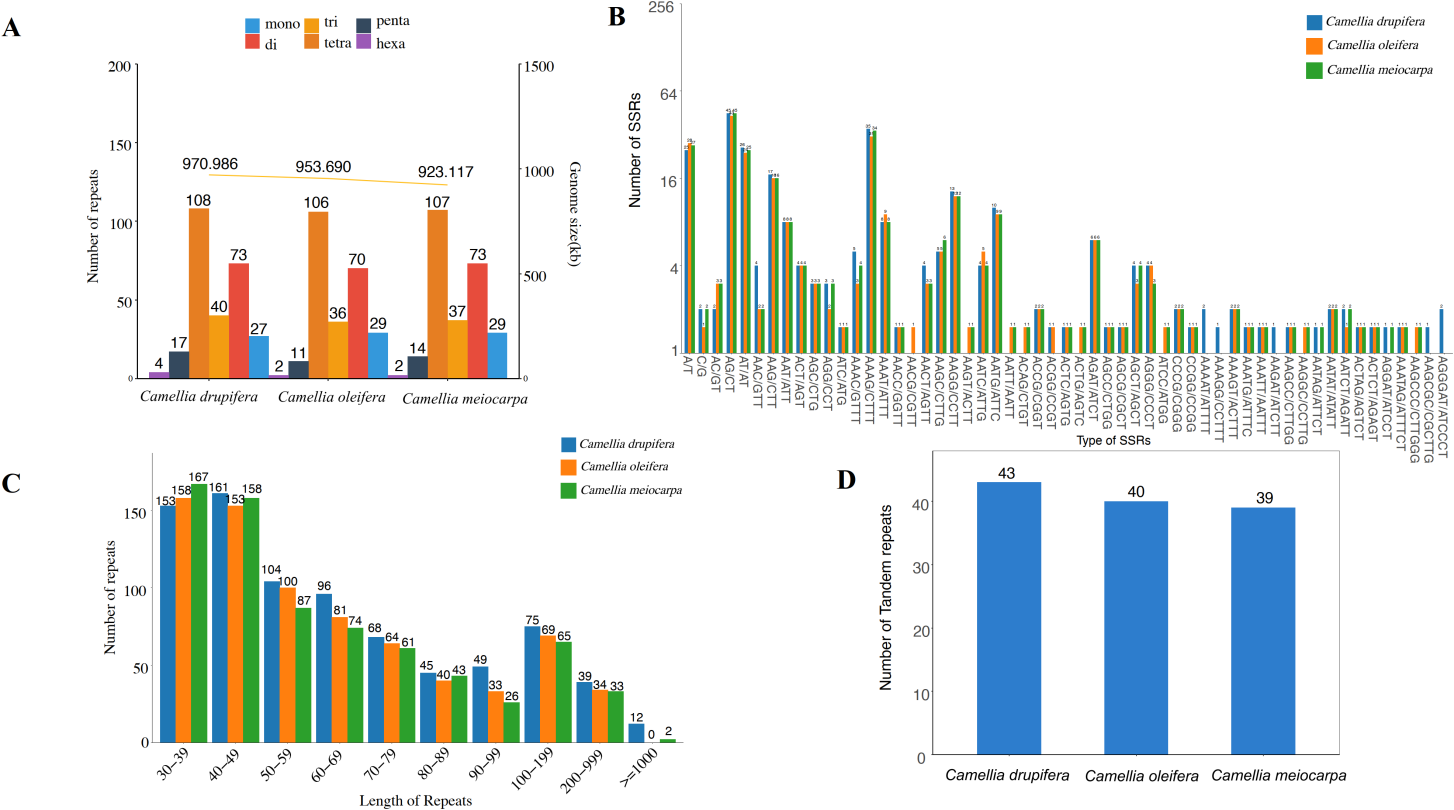
Distribution of repetitive sequences in three main Tea-oil Camellia species’ mitochondrial genome. **(A, B)** the statistics in SSR; **(C)** the statistics in dispersed repeat; **(D)** the statistics in tandem repeats.

Short Sequence Repeats (SSRs) are defined as repeating units varying in length from 1 to 6 base pairs. Our analysis identified a total of 254 SSRs within the mitochondrial genome of *C. oleifera*, including 29 mononucleotides, 70 dinucleotides, trinucleotides 36, tetranucleotides 106, 11 pentranucleotides and 2 hexanucleotides repeats; 262 SSRs in *C. meiocarpa*, including 29 mononucleotides, 73 dinucleotides, 37 trinucleotides, 107 tetranucleotides, 14 pentranucleotides, and 2 hexanucleotides repeats and 269 SSRs in *C. drupifera*, including 27 mononucleotides, 73 dinucleotides, 40 trinucleotides, 108 tetranucleotides, 17 pentranucleotides and 4 hexanucleotides repeats (**Figure 6A**). Tetrameric and dimeric SSRs made up a significant portion of the total, comprising 69.29% in *C. oleifera*, 68.70% in *C. meiocarpa*, and 67.29% in *C. drupifera*. In contrast, pentametric forms were 11, 14 and 17, and only 4, 2, and 2 hexametric SSRs were found among the three main Tea-oil Camellia species (**Figure 6B**).

The four types of dispersed repeats include forward repeats, reverse repeats, complementary repeats, and palindromic repeats. Each type has distinct characteristics and plays different roles in genetic variation and functionality. In **Figure 6C**, we identified 153 (*C. drupifera*), 158 (*C. oleifera*) and 167 (*C. meiocarpa*) repeats in 30-39 bp. The longest repeat, exceeding 1,000 bp, was identified exclusively in *C. drupifera* (12) and *C. meiocarpa* (2). It was observed that the majority of dispersed repeats had lengths under 100 bp. Tandem repeats consist of sequences made up of repeating units that vary in length from 1 to 200 bp, appearing consecutively multiple times. A total of 43, 40 and 39 tandem repeats were identified in *C. drupifera*, *C. oleifera* and *C. meiocarpa* (**Figure 6D**). The total number of dispersed and tandem repeats also showed a strong correlation with the lengths of the mitochondrial genomes.

### 3.4 Analysis of codon usage in the mitochondrial genome of three main Tea-oil Camellia species

To analyze codon usage preferences in the mitogenomes of the three main Tea-oil Camellia species, we computed the relative synonymous codon usage (RSCU), as shown in **Table 2**. An RSCU value greater than one indicates that the codon is preferentially used for its associated amino acid. The mitochondrial DNA of the three main Tea-oil Camellia species encoded all 21 amino acids, with a total of 61 distinct codon types identified. RSCU also showed the tendency to favor A/U bases, and U was used more frequently at the third base of the value of RSCU>1, highlighting a common pattern of codon usage bias across different species. Leucine had the highest number of codons, followed by Serine, while Cysteine had the fewest. Of the codons analyzed, 31 were utilized more often than anticipated (RSCU > 1), while another 31 were employed less frequently (RSCU < 1). Notably, Methionine (AUG) and Tryptophan (UGG) did not show codon preferences, each with an RSCU of 1. Most amino acids, except Methionine (ATG) and Tryptophan (TGG), displayed biases in their codon usage patterns. Most amino acids had at least two codons; for instance, Arginine, Leucine, and Serine each had six codons. This suggests that the protein-coding genes in the three main Tea-oil Camellia species mitogenome exhibit a general codon usage preference.

**Table 2:** Relative synonymous codon usage in three main Tea-oil Camellia species’ mitochondrial genome.

	Codon	*Camellia drupifera*		*Camellia oleifera*		*Camellia meiocarpa*	
		No.	RSCU	No.	RSCU	No.	RSCU
*	UAA	20	1.5789	20	1.5789	20	1.5789
*	UAG	6	0.4737	6	0.4737	6	0.4737
*	UGA	12	0.9474	12	0.9474	12	0.9474
A	GCA	165	0.9593	165	0.9607	165	0.9607
A	GCC	164	0.9535	163	0.9491	163	0.9491
A	GCG	87	0.5058	87	0.5066	87	0.5066
A	GCU	272	1.5814	272	1.5837	272	1.5837
C	UGC	56	0.7368	56	0.7368	56	0.7368
C	UGU	96	1.2632	96	1.2632	96	1.2632
D	GAC	102	0.6036	103	0.6077	103	0.6077
D	GAU	236	1.3964	236	1.3923	236	1.3923
E	GAA	295	1.3288	295	1.3288	295	1.3288
E	GAG	149	0.6712	149	0.6712	149	0.6712
F	UUC	298	0.8949	298	0.8949	298	0.8949
F	UUU	368	1.1051	368	1.1051	368	1.1051
G	GGA	275	1.4686	275	1.4686	275	1.4686
G	GGC	102	0.5447	101	0.5394	101	0.5394
G	GGG	135	0.721	136	0.7263	136	0.7263
G	GGU	237	1.2657	237	1.2657	237	1.2657
H	CAC	61	0.4604	61	0.4604	61	0.4604
H	CAU	204	1.5396	204	1.5396	204	1.5396
I	AUA	228	0.8291	228	0.8311	228	0.8311
I	AUC	235	0.8545	234	0.853	234	0.853
I	AUU	362	1.3164	361	1.3159	361	1.3159
K	AAA	268	1.1729	268	1.1729	268	1.1729
K	AAG	189	0.8271	189	0.8271	189	0.8271
L	CUA	159	0.8712	159	0.8728	159	0.8728
L	CUC	113	0.6192	113	0.6203	113	0.6203
L	CUG	102	0.5589	102	0.5599	102	0.5599
L	CUU	241	1.3205	240	1.3175	240	1.3175
L	UUA	260	1.4247	260	1.4273	260	1.4273
L	UUG	220	1.2055	219	1.2022	219	1.2022
M	AUG	285	1	286	1	286	1
N	AAC	117	0.6802	117	0.6822	117	0.6822
N	AAU	227	1.3198	226	1.3178	226	1.3178
P	CCA	175	1.1532	175	1.1532	175	1.1532
P	CCC	115	0.7578	115	0.7578	115	0.7578
P	CCG	98	0.6458	98	0.6458	98	0.6458
P	CCU	219	1.4432	219	1.4432	219	1.4432
Q	CAA	222	1.5	222	1.5	222	1.5
Q	CAG	74	0.5	74	0.5	74	0.5
R	AGA	178	1.4278	179	1.4358	179	1.4358
R	AGG	92	0.738	91	0.7299	91	0.7299
R	CGA	166	1.3316	166	1.3316	166	1.3316
R	CGC	75	0.6016	75	0.6016	75	0.6016
R	CGG	87	0.6979	87	0.6979	87	0.6979
R	CGU	150	1.2032	150	1.2032	150	1.2032
S	AGC	100	0.6036	100	0.603	100	0.603
S	AGU	171	1.0322	172	1.0372	172	1.0372
S	UCA	189	1.1408	189	1.1397	189	1.1397
S	UCC	159	0.9598	159	0.9588	159	0.9588
S	UCG	146	0.8813	146	0.8804	146	0.8804
S	UCU	229	1.3823	229	1.3809	229	1.3809
T	ACA	132	0.9565	132	0.9565	132	0.9565
T	ACC	143	1.0362	143	1.0362	143	1.0362
T	ACG	80	0.5797	80	0.5797	80	0.5797
T	ACU	197	1.4275	197	1.4275	197	1.4275
V	GUA	198	1.2073	198	1.2073	198	1.2073
V	GUC	119	0.7256	119	0.7256	119	0.7256
V	GUG	144	0.878	143	0.872	143	0.872
V	GUU	195	1.189	196	1.1951	196	1.1951
W	UGG	160	1	161	1	161	1
Y	UAC	75	0.4644	75	0.4644	75	0.4644
Y	UAU	248	1.5356	248	1.5356	248	1.5356

* is termination codon.

We analyzed the PCGs from the mitochondrial DNA of three main Tea-oil Camellia species, determining the GC content for the GC1, GC2, and GC3 of codons. In *C. drupifera*, GC1 ranged from 36.88% to 57.84%, GC2 from 35.51% to 55.86%, and GC3 from 23.93% to 58.38%. For both *C. oleifera* and C. *meiocarpa*, GC1 values were between 36.88% and 57.84%, GC2 ranged from 35.51% to 55.86%, and GC3 varied from 24.79% to 58.41% (**Supplementary Table S6**). On average, the GC content across all three codon positions in these species was under 50%, suggesting a preference for A/T bases and codons ending with A/T in their mtDNA.

**Figure 7:**
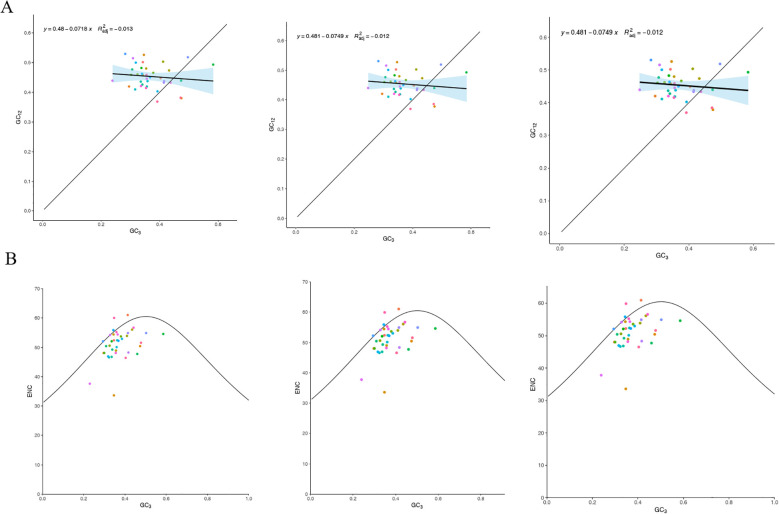
Statistics the correlation of ENC and PCGs among the three main Tea-oil Camellia species. **(A)** GC content of different positions from PCGs and **(B)** ENC-plot against GC3 of mitochondrial genome. From left to right: *C. drupifera, C. oleifera* and *C. meiocarpa*.

The ENC values for the protein-coding genes ranged from 33.62 to 61, with an average exceeding 35, reflecting a relatively weak codon usage bias in the mitochondrial DNA of the three main Tea-oil Camellia species. Moreover, neutrality plot analysis indicated a correlation of 0.1 to 0.2 between GC12 and GC3, with a significance level (P = 0.05) that was lower than anticipated (**Figure 7A**). This implies that the primary factor influencing codon usage bias in the mitochondrial DNA of the three main Tea-oil Camellia species is natural selection pressure.

To investigate the determinants of codon usage patterns in the mitochondrial genomes of the three main Tea-oil Camellia species, we examined 38 PCGs. We calculated the ENC and generated a plot of ENC using GC3 values (**Figure 7B**). Most genes were found below the standard curve, with only a few above, indicating that selection pressure significantly shapes codon preferences.

### 3.5 Gene transfer between chloroplasts and mitochondrial genome in three main Tea-oil Camellia species

**Figure 8:**
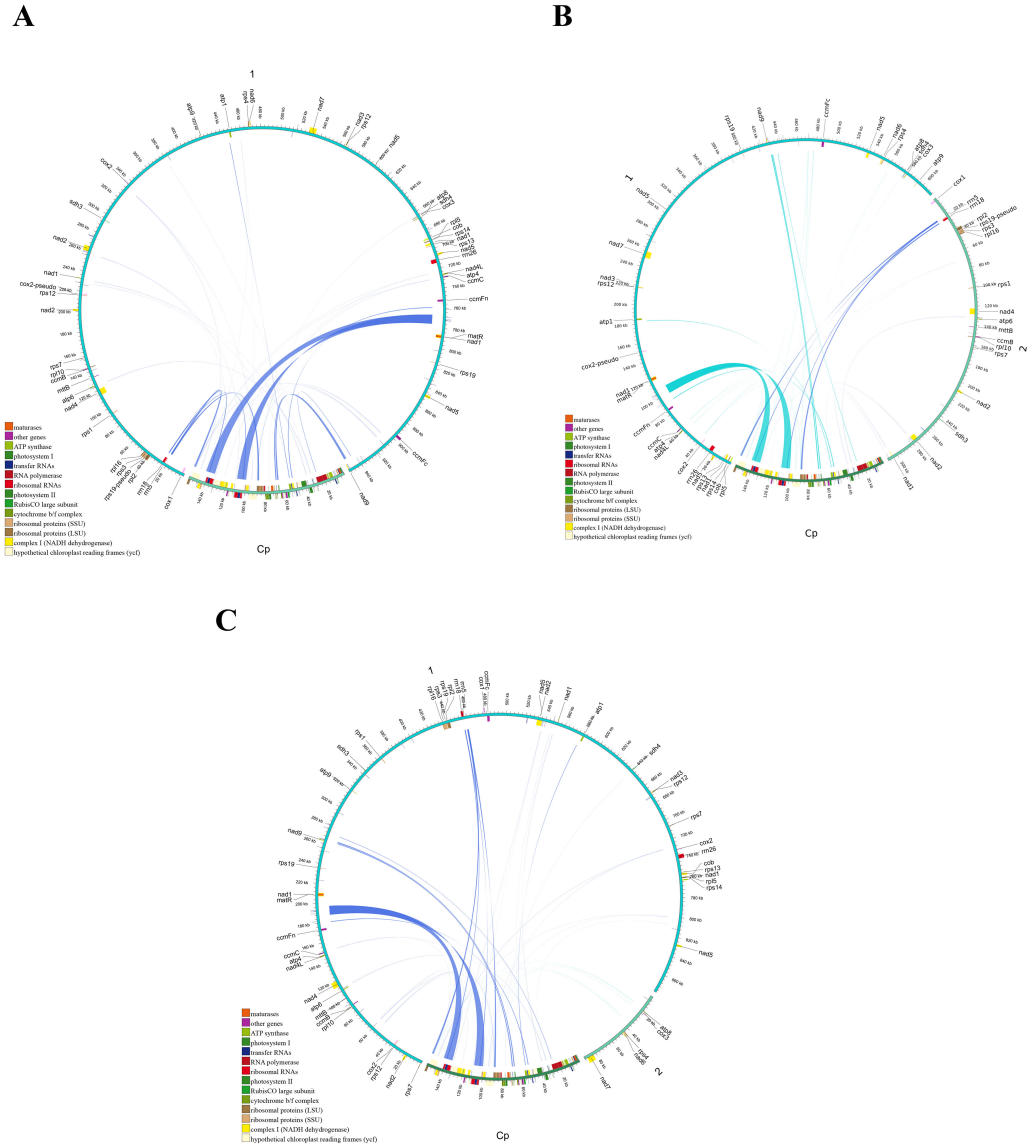
Schematic representation of the chloroplast-to-mitochondrial gene transfer. The chloroplast-to-mitochondrial gene transfer in **(A)** *C. oleifera* **(B)** *C. meiocarpa* and **(C)** *C. drupifera*.

During the evolution of higher plants, the exchange of genetic material between cellular organelles, particularly within mitochondrial genomes, is a common occurrence (Martin and Herrmann, 1998). Mitochondrial plastid sequences (MTPTs) are sequences within mitogenomes that originate from plastids. The homologous fragments between the three main Tea-oil Camellia mitogenome and chloroplast genome were detected and examined. The mitochondrial (mt) genomes of the three main Tea-oil Camellia species were approximately six times larger than their corresponding chloroplast (cp) genomes. A total of 17,141 bp in *C. oleifera*, 16,781 bp in *C. meiocarpa*, and 16,785 bp in *C. drupifera* were derived from chloroplast genomes, accounting for ~1.7–1.8% of their mitochondrial genomes (**Supplementary Table S7**). Within the mt genomes, 31–34 fragments, totaling 27,468–27,802 bp and comprising 17.5–17.76% of the cp genome, were identified as having relocated from the cp genome (**Figure 8**; **Supplementary Table S7**). These fragments exhibited sequence identities exceeding 70%, with alignment lengths ranging from 15 to 174 bp and mismatches between 0 and 42. Additionally, eight complete cp genes (*rrn23, rrn16, psbL, psbJ, psbE, psbF, petG, and petL*) and eight tRNAs (*trnW-CCA, trnA-UGC, trnV-GAC, trnP-UGG, trnM-CAU, trnN-GUU, trnD-GUC, and trnI-CAU*) were observed in both the cp and mt genomes. Moreover, fragmentary sequences of over ten other genes (e.g., *rps12, rpl23, ndhK*, and *atpE*) were also detected in all three species. Interestingly, the migration of cp gene migration occurred unevenly, where the large single copy (LSC) region played a more significant role compared to the inverted repeat (IR) region. Interestingly, MTPTs were predominantly located within repeat-rich regions, suggesting that repeat-mediated recombination might facilitate gene transfer events. This pattern is consistent with observations in other plant species, where organelle genome interactions play a crucial role in mitogenome evolution.

### 3.6 Predict RNA editing sites in three main Tea-oil Camellia species

**Figure 9:**
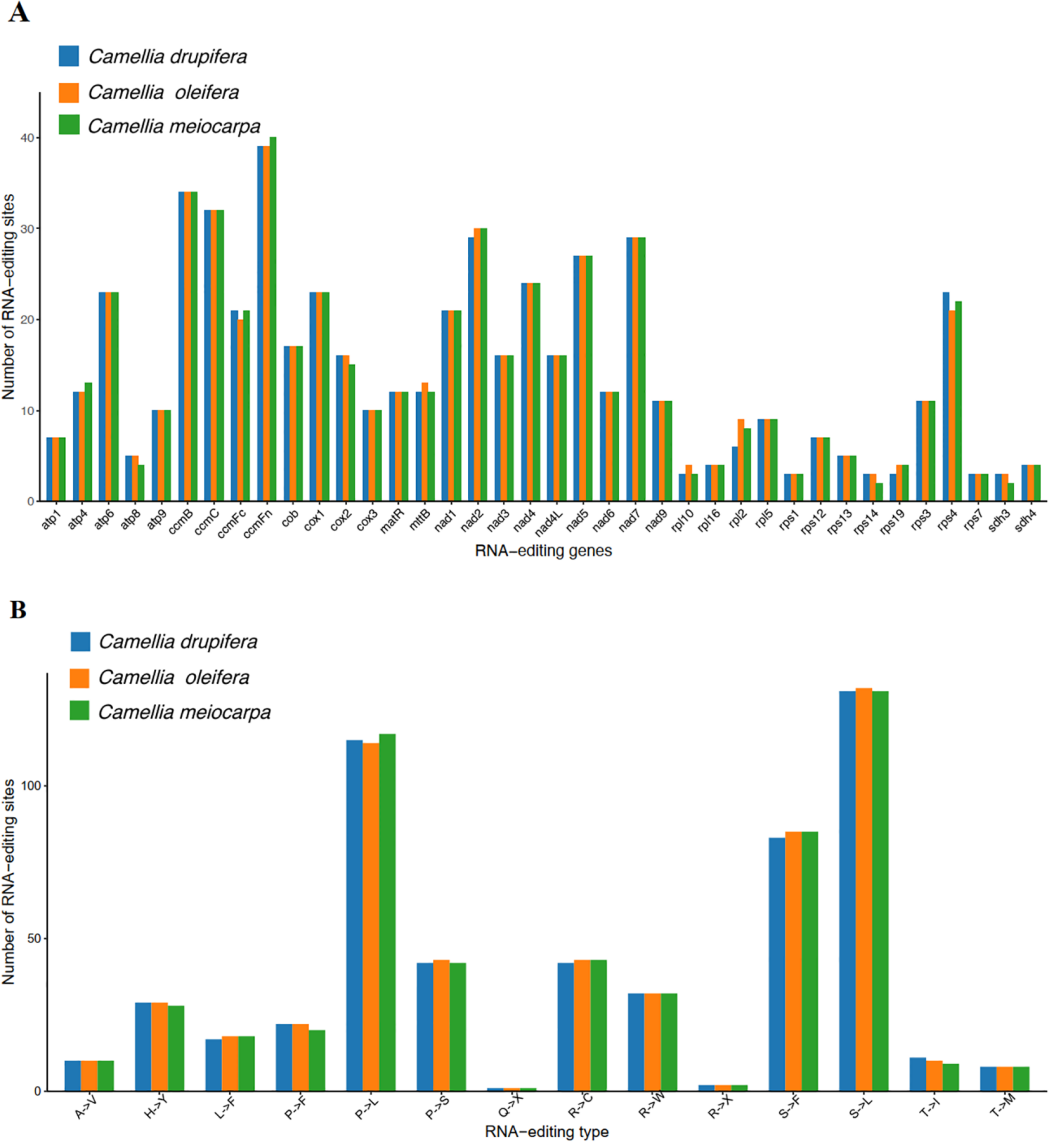
Prediction of RNA editing sites based on the PCGs in three main Tea-oil Camellia species. **(A)** The number of RNA editing sites. **(B)** The number of amino acid transitions.

In all eukaryotes, RNA editing is defined as the process where bases are added, removed, or substituted located in the coding segment of the tRNA (Eisenberg and Levanon, 2018). We identified 549, 546 and 545 RNA editing events across 38 PCGs in the *C. oleifera*, *C. meiocarpa* and *C. drupifera* mitochondrial genomes, respectively (**Figure 9A**). The *ccmFn* gene had the highest number of editing sites, with counts of 39, 40, and 39, followed by *nad2* with 30, 30, and 29. The *rps1*, *rpl10*, *rps14*, *rps19*, *rps7*, *sdh3*, and *sdh4* genes exhibited two to four RNA editing events, associated with ribosomal protein and succinate dehydrogenase functions. Most RNA editing resulted in modifications in amino acids, notably at the first and second base locations, with the latter being more frequently altered (Delaunay et al., 2024). These findings corroborate earlier studies, highlighting substitutions such as Serine (S) to Phenylalanine (F), Proline (P) to Leucine (L), and Serine (S) to Leucine (L), which improve protein stability in the three main Tea-oil Camellia species (**Figure 9B**).

### 3.7 Phylogenetic analysis

**Figure 10:**
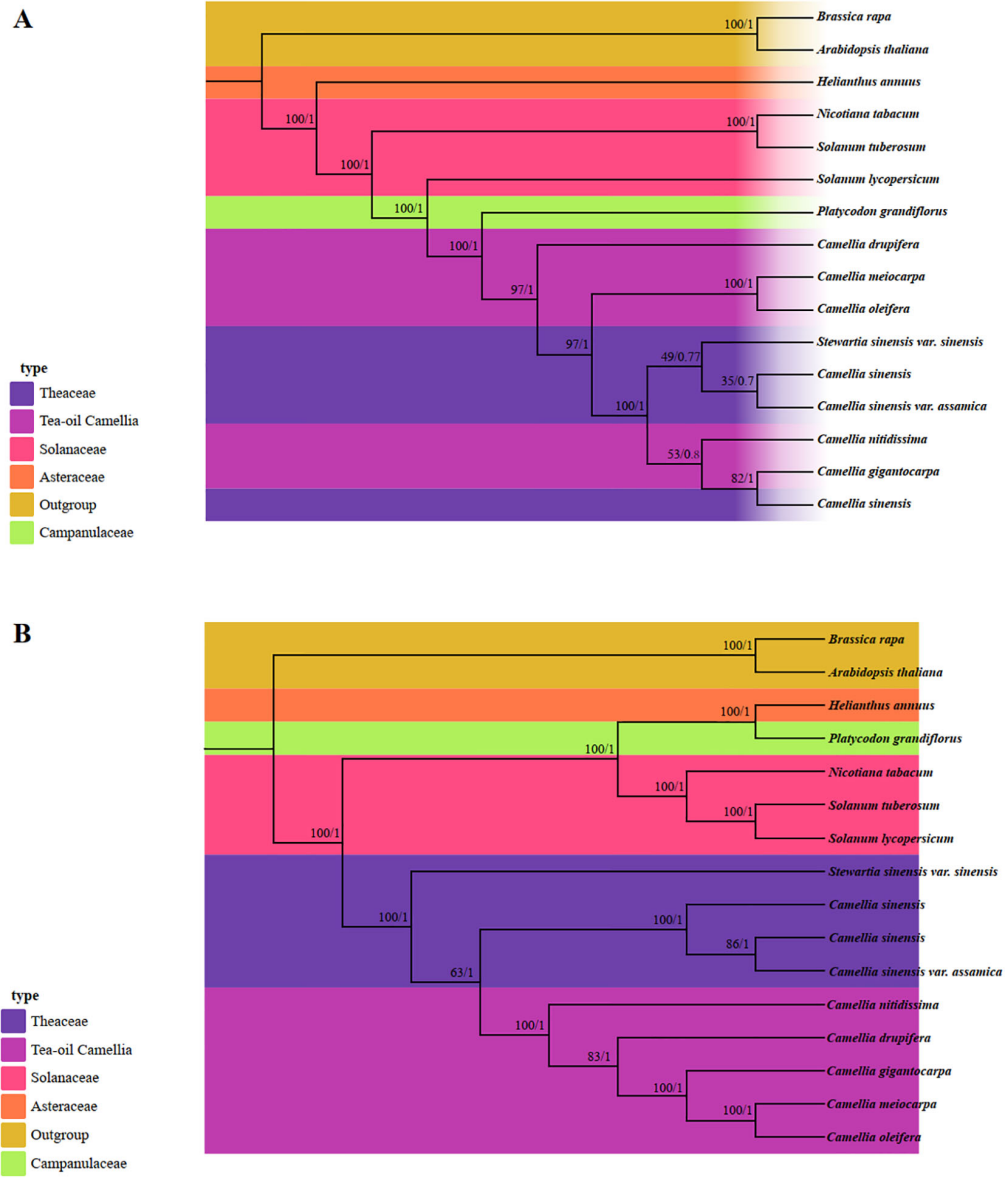
Molecular phylogenetic analysis was conducted by both mitochondrial and chloroplast genomes. **(A)** The mitochondrial phylogenetic tree was analyzed with Maximum Likelihood (ML) and Bayesian Inference (BI) methods. The reliability of the tree was evaluated with bootstrap scores from 1000 replicates, with ML bootstrap support values and BI posterior probabilities indicated at the corresponding nodes. **(B)** The chloroplast phylogenetic tree was constructed by the same species, applying the same methods as those used for the mitochondrial genome-based tree.

In this study, the phylogenetic relationships and taxonomic statuses within the Theaceae family were systematically utilized Maximum Likelihood (ML) and Bayesian Inference (BI) methods for analysis. We utilized the PCGs from 13 published complete mt and cp genomes, along with data from three Tea-oil Camellia species, resulting in a total of 16 species for phylogenetic tree reconstruction (**Supplementary Table S8**). In the mt-based phylogenetic tree (**Figure 10A**), 10 out of 14 nodes showed strong bootstrap support values above 90%, with posterior probabilities of 1. Of these, only 4 out of 8 nodes corresponded to the Theaceae family. The phylogenetic results indicated that *C. drupifera* occupied a basal position, while *C. oleifera* and *C. meiocarpa* formed a sister clade. Additionally, the five Tea-oil Camellia species were not grouped into a single cluster. In contrast, the cp tree (**Figure 10B**) showed higher branch credibility compared to the mt tree. In Theaceae family, the Tea-oil Camellia species were clustered together, with *C. oleifera* and *C. meiocarpa* closely related to *C. drupifera.*

### 3.8 Development of molecular markers in mitochondrial genomes of three main Tea-oil Camellia species

In our study, we aligned and compared mitochondrial sequences from the three main Tea-oil Camellia species, identifying a total of 1,030 SNPs and 460 indels across these regions (**Supplementary Table S9**). The intergenic regions (IGS) contained 1,456 SNPs and indels, while 34 variants were located within gene regions. We tallied the results, and found three IGS *(cox2-trnS-TGA*, *nad6-trnS-TGA-2* and *rpl16-rps1*) existed one hundred more polymorphic sites. These polymorphic sites provide valuable mitochondrial markers for distinguishing between species and hold potential for applications in species identification and population genetics. The result of the design of primers showed in **Supplementary Table S10**.

## 4 Discussion

### 4.1 Structural characteristics and variation of mitochondrial genomes of three main Tea-oil Camellia species

The mitogenome, derived from α-proteobacterial endosymbionts, has experienced numerous transformations, such as structural mutations, rearrangements, gene deletions, and transfers in the genome (Wu et al., 2022). Additionally, the evolutionary path of mitogenomes shows considerable divergence across species, leading to substantial differences among plant species regarding their mitogenomic structures. However, assembling mitogenome in Tea-oil Camellia species presents challenges due to the complex structural alterations and the high prevalence of repetitive sequences (Xiao et al., 2025). In our study, we further confirmed that Tea-oil Camellia species exhibit a multi-branch mitochondrial conformation, which aligns with the mitogenome of the Tea-oil Camellia species (*C. drupifera* and *C. lanceoleosa*) (Liang et al., 2025; Xiao et al., 2025). Each site of the three mitogenomes were reinforced by reads showing ample depth of coverage. Previous study published the mitogenome of *Tea-oil Camellia* species *C. gigantocarpa* (GenBank: OP270590), which was a circular structure (Lu et al., 2022). According to the GenBank data, the mitogenome is a partial genome, which may explain the structural differences compared to the three main Tea-oil Camellia species. In comparison, *C. lanceoleosa*, a diploid relative, exhibits a simpler mitogenome structure, which further highlights the variability in mitogenome architecture within Tea-oil Camellia species (Xiao et al., 2025). This phenomenon was also found in *C. drupifera* (Liang et al., 2025). Our study provides additional evidence suggesting that mitochondrial genomes within *Camellia* species are subject to significant structural complexity, including frequent recombination and rearrangement events, as well as polyploidy, which contribute to the formation of multipartite structures (Wang et al., 2024; Liang et al., 2025). All 24 core genes were annotated, which indicates a complete set based on the coverage of core genes. Therefore, the mitogenomes of *C. oleifera and C. meiocarpa* obtained in this study is regarded as high-quality and complete.

### 4.2 The presence of MTPTs and evolutionary dynamics in three main Tea-oil Camellia

Over time, plants undergo gene transfers between chloroplasts and mitochondria (Stegemann et al., 2012), which can lead to changes in open reading frames (Filip and Skuza, 2021). This genetic exchange has a significant impact on the evolutionary trajectories of plant organelles. The quantity of these MTPTs (mitochondrial plastid DNA sequences) varies widely among different angiosperm species (Wang et al., 2018). Our analysis revealed that the three main Tea-oil Camellia species exhibit varying levels of plastid-derived sequences, with a total of ~17 kb MTPTs detected across all three species, supporting the hypothesis of historical chloroplast-to-mitochondria gene migration. These transferred segments primarily originated from protein-coding genes, tRNA genes, and intergenic regions, suggesting that gene transfer events occurred prior to the divergence of these species. *psaB* and *rrn23*, are functionally conserved, suggesting they play a role in mitochondrial function. Such as *psaB*, supports the hypothesis that ancient organellar gene exchanges have shaped mitogenome evolution in angiosperms (Wang et al., 2007).

Additionally, our study demonstrated a strong correlation between mitogenome size and the abundance of dispersed and tandem repeats, reinforcing the idea that mitochondrial genome expansion is largely driven by repetitive sequences rather than polyploidy (Wang et al., 2021). Repetitive sequences dominated intracellular gene transfer regions, likely facilitating recombination-mediated gene migration between chloroplast and mitochondrial genomes (Scelfo et al., 2024). This observation suggest that repeat sequences act as recombination hotspots, influencing genome rearrangements and stability (Emamalipour et al., 2020).

Beyond their structural impact, repetitive sequences contribute to biological functions, such as gene regulation, DNA repair, and transposable element capture, which may enhance environmental adaptability (Gong et al., 2022). For instance, *C. drupifera*, which contains a higher number of repeats, exhibits stronger heat resistance compared to *C. oleifera* and *C. meiocarpa*. These findings provide new insights into how genome architecture influences species adaptation. By integrating evidence from plastid-to-mitochondria gene transfer, repetitive sequence distribution, and phylogenetic comparisons, this study advances our understanding of mitochondrial genome evolution and organellar interactions in Tea-oil Camellia species.

RNA editing is a complex process that modifies nucleotide sequences at various positions in protein-coding genes, non-coding regions, introns, and tRNA genes (Eddy, 2001; Nishikura, 2016). In the mitogenome of three Tea-oil Camellia species, the *ccmFn* gene had the most editing sites. The *ccmFn*, a key subunit of this complex, is vital for the assembly of holocytochrome, which plays an essential role in electron transport between complex III and complex IV in the respiratory chain (Rayapuram et al., 2008). In the wild-type plant *Hordeum vulgare* subsp. sp*ontaneum*, four *ccmFn* cDNAs were analyzed under high salt stress (Ramadan et al., 2023). A total of 47 nucleotide and amino acid modifications that correspond to ten distinct types of RNA editing. This study raises questions about the mechanisms behind these new editing types and explores the relationship between site C749 and salinity tolerance, which could inform future efforts to enhance plant resilience. The three Tea-oil Camellia species has strong adaptability, and has many good character such as drought-resistance, disease-resistance, enduring salt-aphis and so on. Identifying these RNA editing events in *ccmFn* is crucial for understanding the expression and function of mt genes in the RNA evolution of Tea-oil Camellia.

### 4.3 Adaptive evolution and phylogenetic implications of three Tea-oil Camellia species

The identification of high Ka/Ks ratios in *cox1*, *nad7*, and *rps3* suggests that these genes may be under relaxed selection or undergoing adaptive evolution, particularly in response to environmental stresses. Interestingly, *C. drupifera*, which exhibits greater heat resistance, also harbors the highest number of repetitive sequences, indicating a potential link between genome architecture and stress adaptation. Moreover, we also found the mitochondrial heteroplasmy among them. The presence of multiple mitochondrial genome variants within an individual, is a significant factor in plant evolution (Stewart and Chinnery, 2015; Khachaturyan et al., 2024). While our study did not directly assess heteroplasmy, the observed genome recombination events and gene duplications suggest that Tea-oil Camellia species may exhibit heteroplasmic variation. Further studies utilizing long-read sequencing at the single-cell level could provide deeper insights into the role of heteroplasmy in shaping mitochondrial genome diversity and adaption.

The phylogenetic relationships among three main Tea-oil Camellia species remain contentious and uncertain, as previous analyses have not produced robust or consistent trees (Zhao et al., 2023). In our study, we aimed to reconstruct phylogenetic trees by different datasets and discuss the phylogenetic relationships of the three Tea-oil Camellia species. Based on our results, *C. oleifera* and *C. meiocarpa* in relationship were near with each other while they were far with *C. drupifera*. Generally, species that are closely related evolutionarily tend to have more homologous blocks in common. Homology analysis also support the results of phylogenetic trees. The inconsistency in the *Camellia* phylogeny may be attributed to several factors, including hybridization events, incomplete lineage sorting (ILS), and horizontal gene transfer (HGT), all of which are known to affect organellar phylogenies (Liu et al., 2024c; Park et al., 2025; Yang et al., 2025). To enhance our understanding of the phylogeny and evolution of *Camellia*, it is essential to sequence more mitochondrial genomes, as there is currently a lack of sufficient representative samples. Furthermore, future research incorporating nuclear genome data could further resolve these complex evolutionary relationships.

### 4.4 Molecular markers developed by three Tea-oil Camellia species mtDNA

Molecular markers in the mitogenome offer a robust tool for comparing genetic material variation among populations and individuals improves the accuracy and reliability of plant classification. Previous studies have reported the successful design of such markers within the mitogenome (Shan et al., 2023; Yang et al., 2023). In this study, we identified thousands of variable loci across the three Tea-oil Camellia species. In comparison with the Tea-oil Camellia cpDNA markers we developed, mtDNA markers were more conserved (Liang et al., 2024). These newly developed mitogenome markers have the potential to distinguish among the three Tea-oil Camellia species.

## 5 Conclusion

In our study, we sequenced the mitochondrial genomes of *C. oleifera* and *C. meiocarpa*, revealing a multibranch structure in their mtDNA. Comparative analysis of the three Tea-oil Camellia species showed the unique mtDNA features. Gene transfer between organelles and collinear blocks indicate rearrangement and recombination, yielding significant genetic insights. RNA editing events highlighted that the *ccmFn* gene had the most editing sites, potentially enhancing salt resistance. Phylogenetic analysis further clarified evolutionary relationships among these species, with the developed mitogenome markers showing strong potential for species identification. Our findings also highlight the role of repetitive sequences in mitogenome expansion, the prevalence of inter-organellar gene transfer, and the functional significance of RNA editing events. Overall, these results contribute valuable resources for Tea-oil Camellia breeding, species identification, and evolutionary studies in woody plants.

## Data Availability

The mitochondrial genomes of *C. oleifera* and *C. meiocarpa* are deposited in the GenBank of the National Center for Biotechnology Information (NCBI) repository, accession numbers PQ557234 for *C. oleifera* and PQ557235-PQ557236 for *C. meiocarpa*. All the raw data are available and have been deposited in the NCBI Sequence Reads Archive (SRA) with accession number PRJNA1226724.
